# Aerosol 1,25-dihydroxyvitamin D3 supplementation: A strategy to boost anti-tumor innate immune activity

**DOI:** 10.1371/journal.pone.0248789

**Published:** 2021-03-29

**Authors:** Francesca Bianchi, Michele Sommariva, Valentino Le Noci, Simone Camelliti, Nicoletta Gagliano, Marta Giussani, Andrea Balsari, Elda Tagliabue, Lucia Sfondrini

**Affiliations:** 1 Dipartimento di Scienze Biomediche per la Salute, Università degli Studi di Milano, Milan, Italy; 2 Molecular Targeting Unit, Department of Research, Fondazione IRCCS Istituto Nazionale dei Tumori, Milan, Italy; 3 Laboratory Medicine Unit, Department of Diagnostic Pathology and Laboratory, Fondazione IRCCS Istituto Nazionale dei Tumori, Milan, Italy; University of Pécs Medical School, HUNGARY

## Abstract

**Background:**

1,25-dihydroxyvitamin D3 [1,25(OH)2D3] plays a role in calcium homeostasis but can also exert immunomodulatory effects. In lungs, characterized by a particular immunosuppressive environment primarily due to the presence of alveolar macrophages (AM), 1,25(OH)2D3 has been shown to favor the immune response against pathogens. Here, we explored the ability of aerosolized 1,25(OH)2D3 to locally promote an anti-tumor phenotype in alveolar macrophages (AM) in the treatment of lung metastases.

**Methods:**

Cytotoxicity assay has been used to assess the capability of AM, *in vitro* treated of not with 1,25(OH)2D3, to stimulate NK cells. Sulforhodamine B (SRB) assay has been used to assess the effect of 1,25(OH)2D3 on MC-38 and B16 tumor cells *in vitro* growth. 1,25(OH)2D3 was aerosolized in immunocompetent mouse models to evaluate the effect of local administration of 1,25(OH)2D3 on *in vivo* growth of MC-38 and B16 tumor cells within lungs and on infiltrating immune cells.

**Results:**

*In vitro* incubation of naïve AM with 1,25(OH)2D3 improved their ability to stimulate NK cell cytotoxicity. *In vivo* aerosolized 1,25(OH)2D3 significantly reduced the metastatic growth of MC-38 colon carcinoma, a tumor histotype that frequently metastasizes to lung in human. Immune infiltrate obtained from digested lungs of 1,25(OH)2D3-treated mice bearing MC-38 metastases revealed an increased expression of MHCII and CD80 on AM and an up-modulation of CD69 expression on effector cells that paralleled a strong increased ability of these cells to kill MC-38 tumor *in vitro*.

**Conclusions:**

Together, these data show that aerosol delivery can represent a feasible and novel approach to supplement 1,25(OH)2D3 directly to the lungs promoting the activation of local immunity against cancer.

## Introduction

Emerging evidences have revealed that 1,25(OH)2D3 is not merely a micronutrient playing a crucial role in calcium homeostasis but a pluripotent hormone with immunomodulatory effects. Indeed, the enzyme 1α-hydroxylase, which catalyzes the last and rate limiting step in the synthesis of active 1,25(OH)2D3 from its precursor 25-hydroxyvitamin D3 [25(OH)D3], and the 1,25(OH)2D3 receptor (VDR), which mediates the action of 1,25(OH)2D3, are widely expressed in the body including immune cells [1, 2].

In the oncology field, interest about 1,25(OH)2D3 and 25(OH)D3 has enormously increased after the publication of several studies showing the correlation of 25(OH)D3 serum level and cancer incidence and progression [3]. In cancer patients, high serum 25(OH)D3 concentration, quantified at the time of diagnosis, has been associated with improved survival in different tumor types such as breast and lung cancer [4, 5]. However, results of clinical trials, that explored the effects of 1,25(OH)2D3 supplementation in cancer patients do not seem to be conclusive about the beneficial anti-cancer activity of 1,25(OH)2D3. Several explanations might contribute to explain the variable results of these studies such as polymorphisms in genes involved in 25(OH)D3 absorption, a variable expression of VDR at the tumor bed or the inability of 1,25(OH)2D3 to reach tumor site and exert its biological effects [6].

Direct anti-tumor effects of 1,25(OH)2D3 have been observed in several cancer cell lines where it determined the reduction of cell proliferation, the induction of apoptosis, cell cycle arrest as well as an higher sensitivity to chemotherapeutic agents (review in [7]). Besides these *in vitro* evidences, immunological effects of 1,25(OH)2D3 on the local immune microenvironment have been also observed. In head and neck cancer patients, severe 25(OH)D3 deficiency has been associated with altered intra- and peri-tumoral immune cell infiltration with impaired immune function [8]. Interestingly, when the same patients were supplemented with Vitamin D3 for 3 months, a significant rise in NK cell cytotoxic activity was observed, suggesting that Vitamin D3 treatment could improve anti-tumor immune response by regulating innate immune effector cells. Moreover, the impact of 1,25(OH)2D3 on the tumor microenvironment (TME) might be also related to its anti-inflammatory properties [9, 10]. Moreover, it has been highlighted that low 25(OH)D3 serum levels indicating vitamin D deficiency (VDD) is a risk factor for patients with diffuse large B-cell lymphoma (DLBCL) and follicular lymphoma (FL) and the interference of VDD with ADCC (antibody-dependent cellmediated cytotoxicity), the major mechanism of rituximab action [11, 12]. Epidemiological studies indicate that VDD increases the susceptibility to viral and bacterial respiratory tract infections, notably, upper respiratory tract infections [13–18]. In lungs, 1,25(OH)2D3 regulates the response to pathogens by activating airway epithelial cells, macrophages, dendritic cells and T- and B lymphocytes [10]. For instance, 1,25(OH)2D3 has been shown to enhance the mycobactericidal activity of AM [19], promoting the expression of antimicrobial peptides [20].

Several studies have shown that lungs create a particular permissive milieu for cancer progression where AMs play a key role in maintaining this immune tolerant environment [21]. Balance between inflammatory M1 macrophages and anti-inflammatory M2 macrophages at tumor site has been strictly related with cancer progression [22]. In human macrophages, 1,25(OH)2D3 regulates phagocytosis [23], activates superoxide synthesis [24], and induces expression of cathelicidin [25]. M1 macrophages are able to kill B cell lymphoma cells via vitamin D–mediated induction of the cathelicidin peptide, whereas M2 macrophages and M2-like tumor-associated macrophages (TAMs) exhibit an altered vitamin D metabolism, resulting in a reduced production of cathelicidin [26].

Since 1,25(OH)2D3 exerts a stimulatory effect on AM and activation of the vitamin D signaling pathway can restore tumoricidal effector mechanisms of TAMs, its local supplementation could promote an anti-tumor phenotype of these immune cells.

Bronchial and bronchoalveolar tumors are accessible via the endobronchial space by inhalation that allows to reach high local drug concentration, avoiding systemic side-effects. Previously it has been demonstrated that repeated aerosol administration of immune stimulants, as TLR agonists, promotes the activation of NK cells [27] against melanoma metastases through a direct stimulation of AM. Furthermore, it has been recently observed that antibiotic aerosolization, by modulating lung microbiota, is an effective strategy to promote activation of pulmonary macrophages and NK cells against cancer [28]. Inhalation of 1,25(OH)2D3 and 25(OH)D3 have been recently investigated to induce neonatal lung maturation [29] without the occurrence of systemic side effects, such as the hypercalcemia usually associated to its parenteral and oral administration. However, to the best of our knowledge, no study investigated local delivery of 1,25(OH)2D3 to stimulate an antitumor effect in lungs.

In the present work, we evaluated whether 1,25(OH)2D3 enhances the anti-tumor activity of effector cells *via* the activation of AM and the therapeutic activity of 1,25(OH)2D3 when administered by aerosol to treat of lung metastases. Our data also could open new perspective to consider the relationship between 1,25(OH)2D3 deficiency and COVID-19 affections and severity.

## Materials and methods

### Cell lines and reagents

MC-38 mouse colon cancer cells and B16 murine melanoma cells YAC-1 murine lymphoma (ATCC, American Type Culture Collection, Rockville, MD, USA) were routinely maintained at 37°C in a 5% CO_2_ atmosphere in DMEM medium and RPMI medium (Thermo Fisher Scientific Inc., Waltham, MA, USA) supplemented with 10% fetal bovine serum (Thermo Fisher Scientific) and 2 mM glutamine (Sigma-Aldrich, St. Louis, MO, USA).

Cell line was authenticated by the Fragment Analysis Facility at Fondazione IRCCS–Istituto Nazionale dei Tumori (Milan, Italy) using the GenePrint 10 System (Promega, Madison, WI, USA) and cultures were regularly tested for Mycoplasma by using the mycoAlert Plus Kit (Lonza Group Ltd, Basel, Switzerland).

### Cells isolation and culture

Bronchoalveolar lavage (BAL) was performed in euthanized C57BL/6 mice by 5 intratracheal instillations of 1 ml PBS, containing 0.5% BSA and 2 mM EDTA, followed by gentle aspiration. The recovered BAL fluid was then centrifuged. The resulting cells were plated for 3 h at 37 °C in complete medium on 48-well plates to allow macrophages to adhere to the culture plates, and non-adherent cells were recovered by washing with PBS. After 24 h adhered macrophages were treated with 1,25(OH)2D3 (100 nM) for 24 h.

### Isolation of lung suspensions

Isolation of lung immune cells was performed as described [27]. Briefly, lungs were digested in DMEM medium containing collagenase (300 U/ml) and hyaluronidase (100 U/ml) (Stemcell Technologies, Vancouver, Canada) for 1 hr at 37°C. Cell suspensions were then filtered through 70-μm cell strainers and, after lysis of red blood cells, were directly stained for flow cytometry or plated to separate adherent and non-adherent cell fractions as described [30].

### Quantitative PCR analysis

RNA was isolated using QIAzol (QIAGEN, Hilden, Germany) and Direct-zoltm RNA MicroPrep (Zymo Research, Irvine, CA, USA) from adherent cells according to the manufacturer’s instructions. Reverse transcription was performed using a High-Capacity RNA-to-cDNA Kit (Applied Biosystems-Thermo Fisher Scientific). Real-time PCR was performed using TaqMan^®^ Fast Universal PCR Master Mix (Applied Biosystems-Thermo Fisher Scientific) and SDS 2.4 on a 7900HT Fast Real-Time PCR System (Applied Biosystems-Thermo Fisher Scientific), as previously described [31]. The following TaqMan^®^ gene expression assay (Applied Biosystems-Thermo Fisher Scientific) was used in Real Time PCR analyses: *IL12* (assay ID: Mm00434169_m1; best coverage assay to recognize interleukin 12a gene). The expression of this gene was normalized to *B2m* (assay ID: Mm00437762_m1). PCR data were analyzed using the 2-ΔCt method.

### Flow cytometry

To analyze immune infiltration, lung suspensions were stained for 30 min at 4°C using following directly conjugated antibodies: CD45 BV786 (BD Horizon, clone 30-F11); F4/80 BV650 (BD OptiBuild, clone: T45-2342); CD11c BV605 (BD Horizon, clone HL3); CD80 PE (BD Pharmingen, clone 16-10A1); MHC II BB700 (BD OptiBuild, clone 2G9); CD69 PE (BD Pharmingen, clone H1.2F3) and LIVE/DEAD FVS780 BD HorizonFixable Viability Stain 780). A purified rat anti-mouse CD16/CD32 MAb (eBiosciences, clone 93) was used to block non-specific binding to mouse Fc receptors.

The cells were analyzed using a FACS Celesta flow cytometer (BD Biosciences) and FlowJo software (TreeStar). All analyses were performed gating on CD45+ live cells after doublet exclusion.

### Growth in vitro assay

The degree of proliferation of MC-38 cells and B16 cell following treatment was evaluated by measuring relative 2D cell growth by sulforhodamine B (SRB) assay [32]. Briefly, cells were seeded at a density of ~2.5x103 (cells per 100 μl/well in 96-well plates in the appropriate growth medium and allowed to attach for 24 h before treatment. The medium was replaced with fresh medium containing serial dilutions of 1,25(OH)2D3 (50–6.3 nM) in a volume of 100 μl/well, or left untreated. The plates were incubated at 37°C for an additional 72 h. After incubation, 100 μl of 10% trichloroacetic acid (TCA) was added to each well and plates were incubated for 1 h at 4°C. Afterwards, the medium was removed and cells were washed 5 times with deionized water. Following overnight (ON) airdrying, 100 μl of SRB solution (0.4% (w/v) in 1% acetic acid) was added to each well. After incubation for 15 min plates were washed 5 times with 1% acetic acid and air-dried. The protein-bound dye was solubilised with a 10 mM Tris-base solution (pH 10.5). Optical density was determined on an ELISA microplate reader (Bio-Rad Laboratories, Hercules, CA, USA). Differences were considered to be significant at p < 0.05.

### Cytotoxicity assays

Two different cytotoxic assays were performed. In the first test described in the paragraph 3.1, AM obtained from BAL of C57BL/6 healthy mice were cultured in presence or not of 1,25(OH)2D3 100 nM for 24 h then were extensively washed and co-cultured with purified splenic NK cells at 1:1 ratio for 24 hrs. NK cells were then harvested and used for the cytotoxic assay against ^51Cr^ YAC-1 cell line at an effector:target ratio of 50:1.

In the second test described in the paragraph 3.4 the suspension obtained from lung digestions were plated for 2 h at 37 °C to separate adherent cells from non-adherent cells, which contain T and NK cells. Then, recovered non-adherent cells fraction was used to test the ability to kill MC-38 target cancer cells at an effector:target ratio of 50:1. In both assays, the radioactivity of the supernatant (80 μl) was measured as described [33].

### Mice and experimental protocols

Female C57BL/6 mice, aged 6–8 weeks (Charles River, Wilmington, MA, USA), were maintained in laminar flow rooms at constant temperature and humidity, with food and water given ad libitum. The experiments were approved by the Ethics Committee for Animal Experimentation of Fondazione IRCCS Istituto Nazionale dei Tumori, Milan. To evaluate the effects of aerosolized 1,25(OH)2D3 on tumor metastatization, mice were treated with 1,25(OH)2D3 48 h interval starting 4 days after they were i.v. injected with 5x10^5^ MC-38 colon carcinoma cells or with 3x10^3^ B16 murine melanoma cells and continuing throughout the experiment. Aerosolization was performed using a tower inhalation system (IES 306 Inhalation Towers EMMS). 1,25(OH)2D3 suspension (50 ng/5 ml saline) was placed in the nebulizer (Aeroneb Lab Micropump Nebulizer) and used to treat groups of 6 mice by exposure to aerosol for 10 min [28].

To evaluate the effects of *in vivo* blocking of IL-12, mice were treated with anti-IL-12 antibody or isotype control (ip treatment of 150 μg at the first day of 1,25(OH)2D3 treatment, followed by intranasal administration of 70 μg, two times/week for 3 weeks), starting 4 days after i.v. injection with 5x10^5^ MC-38 cells and were treated or not with aerosolized 1,25(OH)2D3 as above.

Mice were weighed and inspected for any sign of sufferance twice weekly and euthanized at day 21 after tumor injection. Using forceps, lungs were pry apart into individual lobes and tumor individual nodules were enumerated with a cell counter on each lung blindly by two operators, with the help of a lamp and a magnifying glass; total for all lobes was also noted. B16 nodules clearly show up black, with occasional amelanotic, gray/white nodules. The quantification of the number of lung metastatic foci of MC38 cancer cells was quite difficult since they appeared diffused and spread. In the case of MC38 metastases, one mice belonging to the saline-treated group showed foci too broad and diffuse to be counted, then lung weight was also evaluated.

### Statistical analysis

Differences among groups were compared using a two-tailed unpaired Student’s t-test and considered significant at p ≤ 0.05. All analyses were performed using GraphPad Prism version 5.0 for Windows (GraphPad Software, CA, USA).

## Results

### *In vitro* 1,25(OH)2D3-primed alveolar macrophages increase NK cells cytotoxic activity

**Fig 1:**
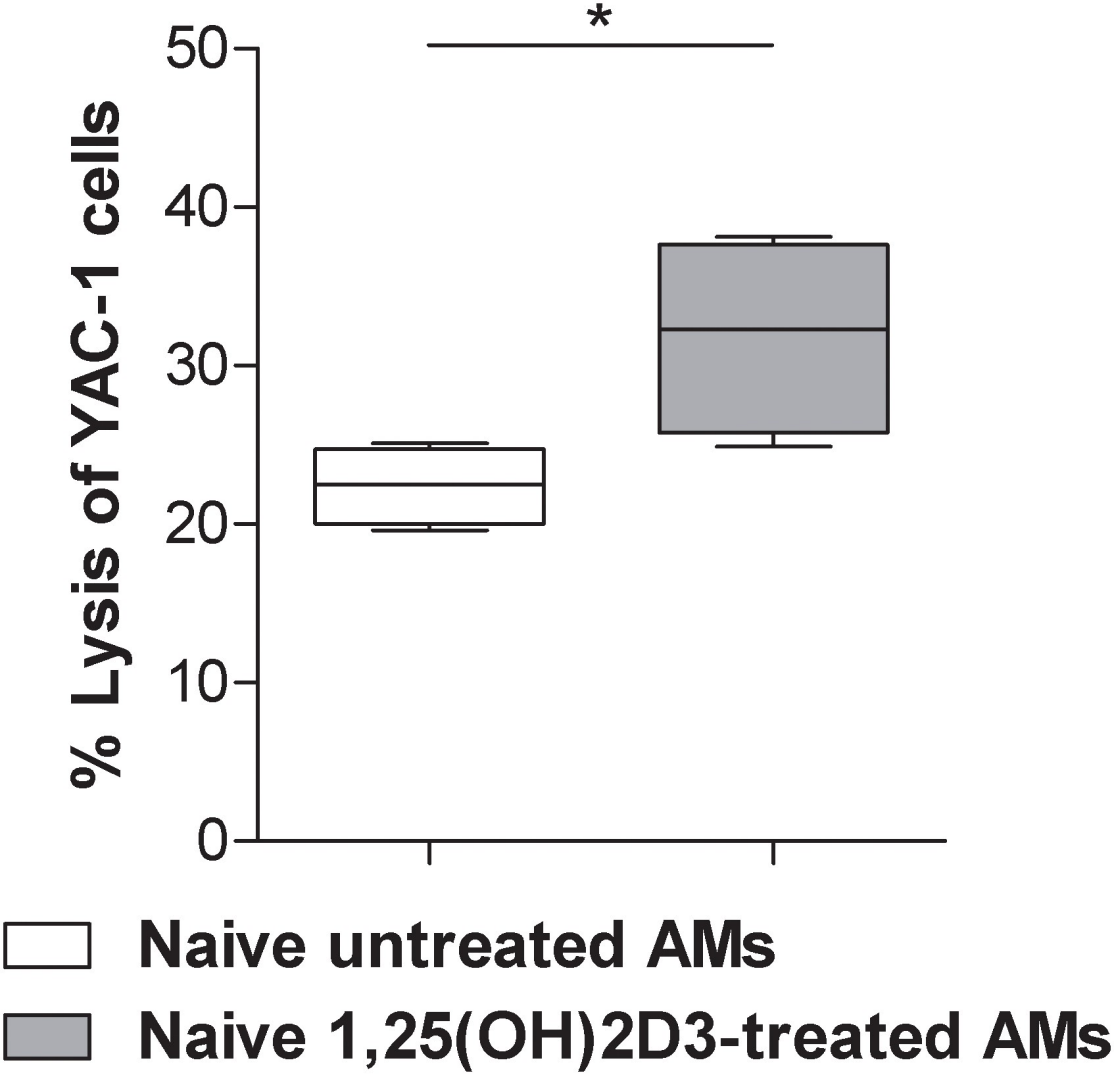
1,25(OH)2D3 increased the ability of murine alveolar macrophages to stimulate in vitro NK cell cytotoxicity. NK cells, from spleen of C57BL/6 healthy mice, co-cultured with 1,25(OH)2D3-pretreated lung macrophages significantly increased the percentage of lysis of YAC-1 cells, as compared to NK cells co-cultured with untreated lung macrophages. Unpaired t test; *p<0.05.

It has been previously demonstrated that AM can activate NK cell cytotoxic activity *in vitro* and *in vivo* [30]. To determine the effect of 1,25(OH)2D3 exposure on the priming ability of AM, adherent cells obtained from BAL of C57BL/6 healthy mice, which primarily contains AM (S1 Fig), were cultured in presence or not of 1,25(OH)2D3 (100 nM) for 24 h. Macrophages were extensively washed and co-cultured with purified splenic NK cells. NK cells were then harvested and used for the cytotoxicity assay against NK-sensitive ^51Cr^ YAC-1 cell line. NK cells co-cultured with 1,25(OH)2D3-pretreated lung macrophages significantly increased the lysis of YAC-1 cells, as compared to NK cells co-cultured with untreated lung macrophages (Fig 1). These *in vitro* results indicate that treatment of AM with 1,25(OH)2D3 results in an increased ability to stimulate NK cell cytotoxicity.

### *In vivo* and *in vitro* anti-tumor effects of 1,25(OH)2D3

To investigate the therapeutic effect of 1,25(OH)2D3 local administration against a growing tumor in the lung, we explored the possibility to deliver 1,25(OH)2D3 directly to the lungs by aerosol administration. Considering that lung metastases most frequently originate from intestinal cancer, we utilized MC-38 colon carcinoma experimental metastasis model. Two groups of C57BL/6 mice were injected i.v. with MC-38 cells and treated with aerosolized 1,25(OH)2D3 or saline.

**Fig 2:**
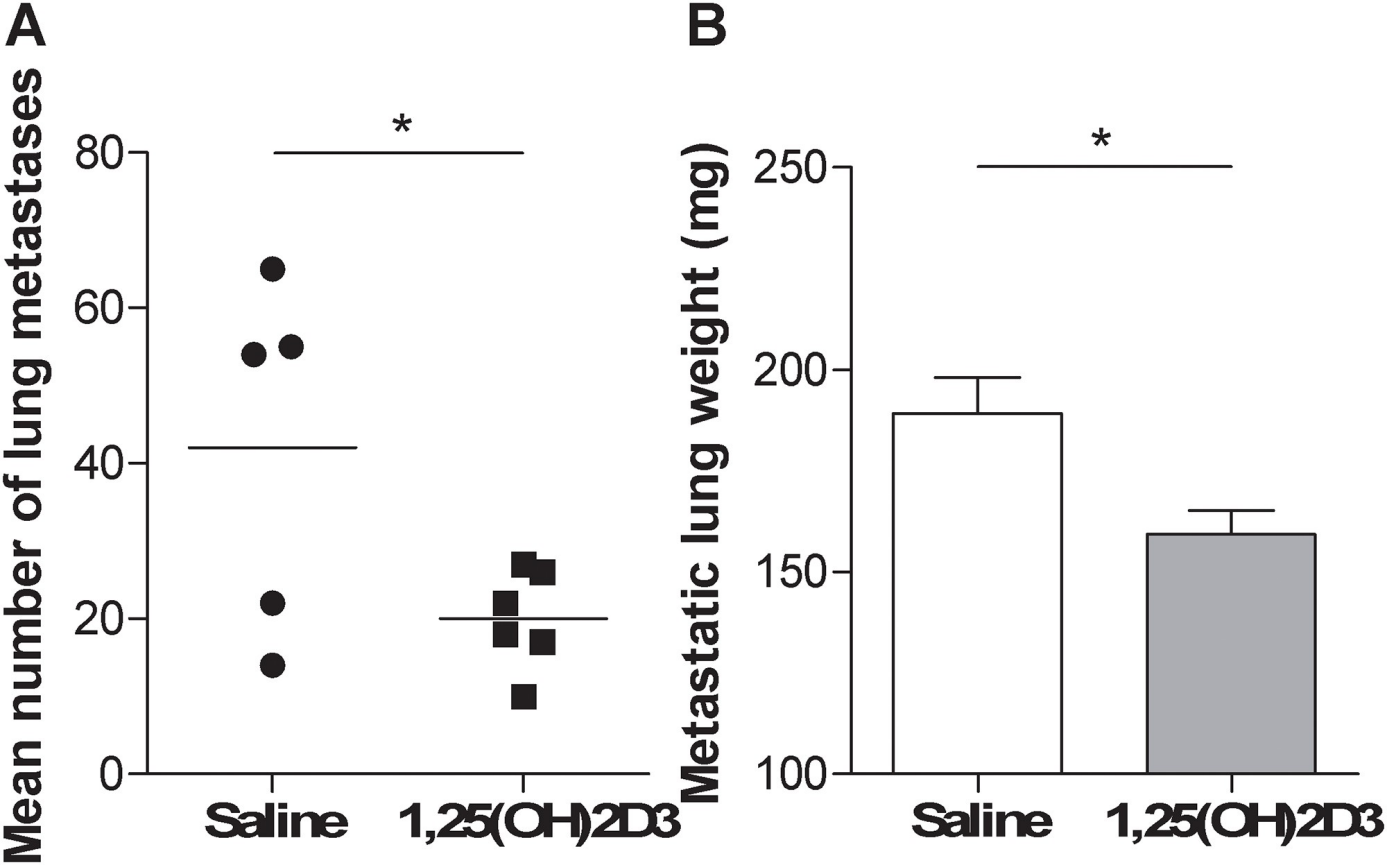
Aerosolized supplementation of 1,25(OH)2D3 reduced metastatic tumor growth in the lung. MC-38 colon cancer cells were injected in C57BL/6 mice and mice were treated with aerosolized saline or 1,25(OH)2D3 as described in Materials and Methods. At the end of the experiment was evaluated (A) in saline-treated group (n = 5) and in 1,25(OH)2D3-treated group (n = 6) mean number of lung metastases and (B) in saline-treated group (n = 6) and in 1,25(OH)2D3-treated group (n = 6) metastatic lung weight. Unpaired t test; *p<0.05.

1,25(OH)2D3 aerosolization significantly reduced the number of metastatic foci in lungs, as revealed by analyzing the number of macroscopic metastasis and the lung weight (Fig 2(A) and 2(B)). A reduction in the number of macroscopic lung metastases, even if not at a significant level, was observed in mice injected i.v. with B16 melanoma cells and treated as above (Mean number of lung metastases: 43.4 ± 13.1 in saline-treated *versus* 22.4 ± 7.2 in 1,25(OH)2D3-treated mice), suggesting the ability of 1,25(OH)2D3 in reducing the growth lung metastases also in a different tumor histotype.

**Fig 3:**
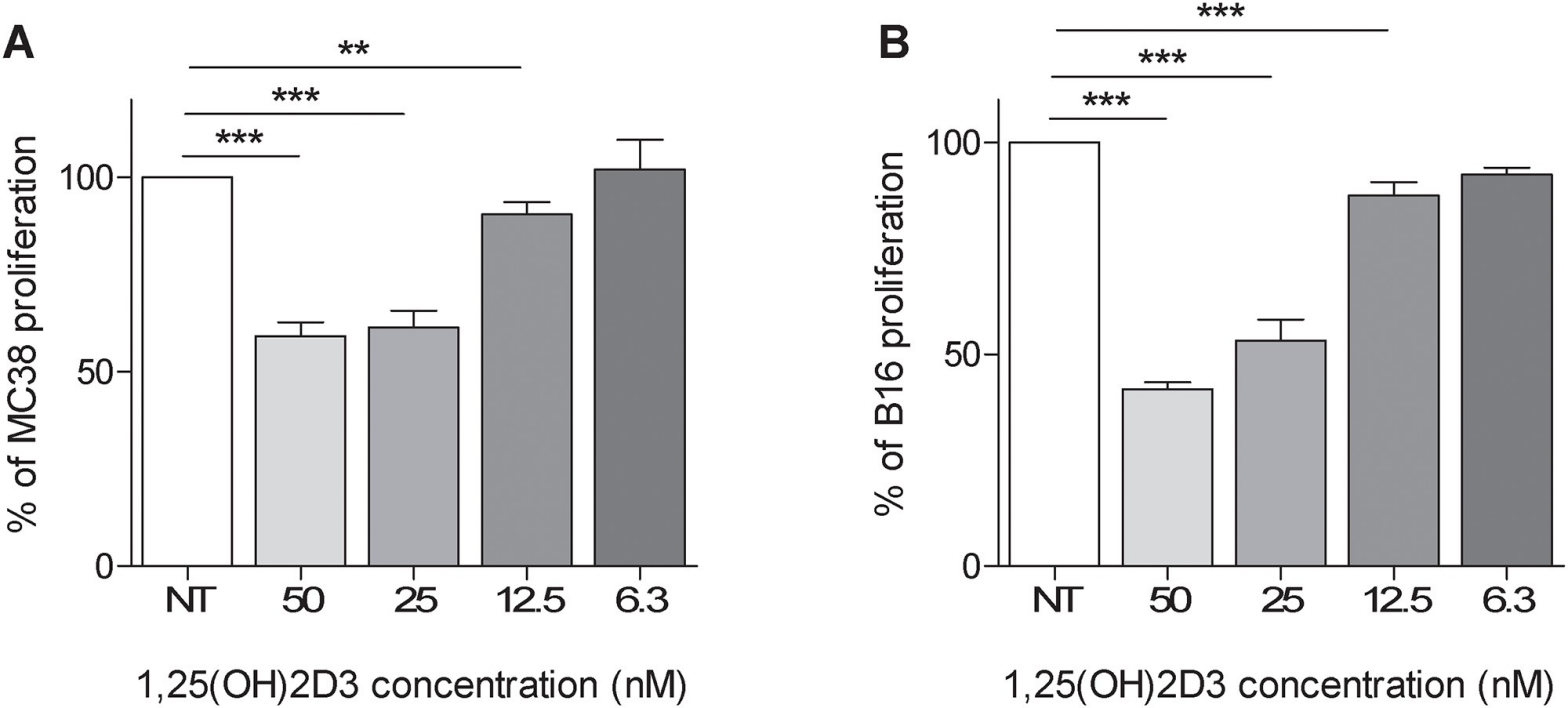
Anti-proliferative *in vitro* activity of 1,25(OH)2D3 against cancer cells. MC-38 colon cancer cell (A) and B16 melanoma cells (B) were exposed to different concentration of 1,25(OH)2D3 for 72 h. Cells proliferation was evaluated by SRB assay at the end of the experiment as the optical density (OD) of 1,25(OH)2D3-treated cells/ the optical density (OD) of 1,25(OH)2D3-untreated cells *100; bars represent the mean of cells proliferation ± SD; Unpaired t test ** p<0.005; ***p<0.0001.

Because 1,25(OH)2D3 induces the differentiation, cell cycle arrest, and apoptosis in different colon and colorectal carcinoma cells [34], we evaluated the effects of 1,25(OH)2D3 on MC-38 cell growth *in vitro* (Fig 3(A)). A significant decrease in cell growth compared to untreated cells was observed when using 1,25(OH)2D3 at concentrations ranging from 50 to 12,5 nM. Concentrations of 1,25(OH)2D3 below 12.5 nM did not exert any effect. Similar results were observed on the growth of B16 melanoma cells (Fig 3(B)).

This data suggest that 1,25(OH)2D3 administration reduced *in vitro* proliferation of MC-38 and B16 cancer cells at concentrations similar to those utilized *in vivo* experiments. Therefore, 1,25(OH)2D3 direct effects on cancer cells can partially explain the observed *in vivo* antitumor activity.

### Effect of aerosolized 1,25(OH)2D3 on tumor infiltrating immune cells

**Fig 4:**
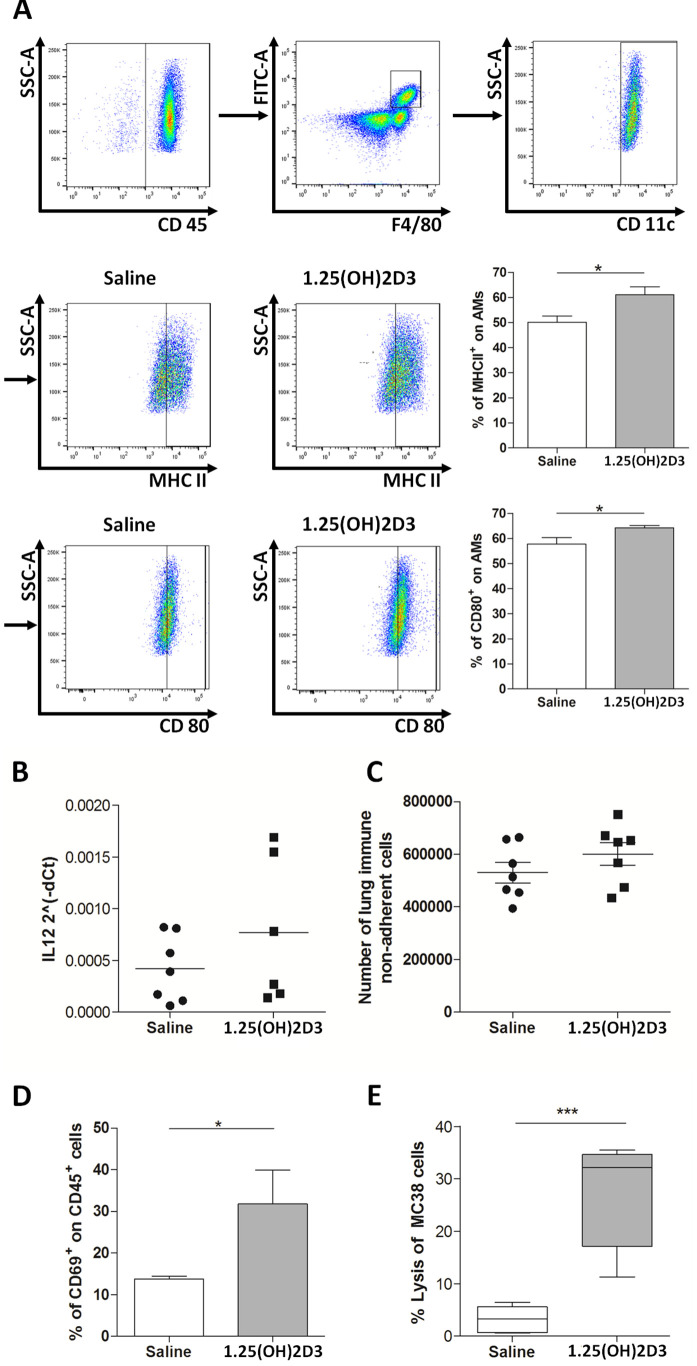
1,25(OH)2D3 aerosolization induces activation of innate immune effectors in the lung. Lungs were recovered from tumor-bearing mice treated with 1,25(OH)2D3 or saline. Flow cytometry analysis of the expression of MHCII and CD80 maturation markers on AM of lungs digestion (A). Real time PCR analysis of IL-12 mRNA expression in adherent fraction from lung suspensions (B); count of cells in the non-adherent fraction containing effector cells from lung suspensions (C); flow cytometry analysis of CD69 activation marker on the non-adherent fraction (D); percentage of MC-38 lysated cells evaluated by *in vitro* cytotoxic assay performed with non-adherent fractions from lung suspensions (E); Unpaired t test; ***p<0.0001.

The *in vivo* effect of 1,25(OH)2D3 on lung immune tumor-infiltrating cells, obtained by enzymatic digestion of lungs of MC-38 tumor bearing-mice treated with aerosolized 1,25(OH)2D3 or saline, was analyzed by flow cytometry. AM from 1,25(OH)2D3-treated group revealed an up-modulation of MHCII and CD80 maturation markers (Fig 4(A)).

At the same time, suspensions of lung digestions were plated for 2 h at 37 °C to separate adherent cells, mostly containing macrophages, from non-adherent cells (T and NK cells). An increased expression of IL-12 mRNA level was observed in adherent cells fraction from tumor-bearing mice treated with aerosolized 1,25(OH)2D3 compared to that treated with saline (Fig 4(B). Count of cells in the non-adherent fraction did not reveal any significant change between saline- or 1,25(OH)2D3-treated mice (Fig 4(C)), although flow cytometry analysis showed an increased expression of CD69 activation marker on the fraction obtained from 1,25(OH)2D3-treated mice (Fig 4(D)). Accordingly, *in vitro* cytotoxic assay, using non-adherent cells fraction from tumor-bearing mice treated with aerosolized 1,25(OH)2D3, showed a strong increase in the ability to kill MC-38 target cancer cells compared to those obtained from saline-treated mice (Fig 4(E)).

To ascertain whether the anti-tumor activity of aerosolized vitamin-D relies on its ability to stimulate immune cells, mice were i.v. injected with MC-38 cells and intranasally administered with anti-IL-12 antibody or isotype control immediately before 1,25(OH)2D3 aerosolization. Two groups of mice were treated with anti-IL-12 antibody or isotype control without 1,25(OH)2D3. At the end of the experiment, an increased lung metastases number was observed in mice treated with anti-IL-12, as compared to isotype control-treated mice (mean number of lung metastases ± SEM: 78.5 ± 15.2 in IL-12-treated versus 65.2 ± 12.0 in isotype-treated mice). No significant differences were observed in the presence of anti-IL-12 administration between 1,25(OH)2D3- treated and untreated mice (mean number of lung metastases ± SEM: 93.0 ± 7.0 in IL-12 plus 1,25(OH)2D3-treated *versus* 78.5 ± 15.2 in IL-12-treated mice), suggesting the relevance of the involvement of this immune cytokine in mediating the anti-tumor 1,25(OH)2D3 effect.

## Discussion

The data presented in the present study reveal that 1,25(OH)2D3 aerosolization may represent a strategy to awaken innate immune system and counteract tumor growth in lungs.

Interest about the role of 1,25(OH)2D3 in cancer originates from retrospective observations correlating low serum level of its precursor 25(OH)D3, used as index of vitamin D status in body, with increased risk of developing multiple malignancies [3]. However, evidences in support of a causal effect of 25(OH)D3 on cancer are still limited [35, 36]. Regarding cancer in the lung, a meta-analysis of available clinical data showed an inverse association between circulating levels of 25(OH)D3 and risk of developing tumor [5, 37, 38]. However, a Cochrane Systematic Review failed to reveal evidences of preventive efficacy of vitamin D supplementation (administered as Vitamin D2, or Vitamin D3, or 25(OH)D3, or 1,25(OH)2D3) for any malignancy [39].

Patients with low 25(OH)D3 levels are usually diagnosed with higher cancer mortality, suggesting that 1,25(OH)2D3 might also protect patients against advanced stage of cancer [3]. Retrospective observational studies on the effect of vitamin D supplementation in cancer patients also provide evidences of potential anti-cancer capability of 1,25(OH)2D3 even if the results of these studies not allow to reach a consensus about the opportunity to prescribe vitamin D supplementation in cancer patients. These data are also supported by few randomized clinical trials that explored the potential benefit of vitamin D administration in cancer patients. Recently, results of a randomized, placebo-controlled trial indicated that supplementation with vitamin D3 did not result in a lower incidence of invasive cancer [36]. However, the metabolism of 25(OH)D3 is dysregulated in many types of cancer, conferring resistance to the potential antitumorigenic effects of vitamin D3 intake [40].

Although these clinical studies seem contradictory in defining the protective role of 1,25(OH)2D3 against tumor appearance and growth, preclinical *in vitro* and *in vivo* studies in various cancer models revealed that 1,25(OH)2D3 can interfere with many cellular pathways leading to the inhibition of proliferation, induction of cell apoptosis and differentiation, promotion of autophagy, as well as suppression of metastasis and angiogenesis (review in [7, 40]). Moreover, 1,25(OH)2D3 possesses potential anti-tumor effect acting as regulator of tumor metabolism in prostate and breast cancer cells [41, 42].

In agreement with these data, we observed that 1,25(OH)2D3 significantly impaired *in vitro* growth of MC-38 and B16 tumor cells in a dose-dependent manner. Therefore, the reduction of tumor growth induced by aerosolized 1,25(OH)2D3 can in part be due to a direct effect on cell proliferation.

As a support, to explore the concentrations of 1,25(OH)2D3 reached in the alveoli after aerosolization, C57BL/6 mice were treated with aerosolized 10 nM 25(OH)D3 or saline and BAL have been recovered within 30 minutes. have been performed. Quantitative determination of 25(OH)D3 by Elecsys Vitamin D total II assay on the Cobas e 801 Immunoanalyzer (Roche Diagnostics) in two independent pilot experiments revealed 7.3 ng/ml (SEM 1.6, n = 2) and 6.3 ng/ml (SEM 3.5, n = 2), suggesting that at least 60% of aerosolized 25(OH)D3 is reached in BAL.

*In vivo* neutralization of IL-12 abrogated 1,25(OH)2D3 antitumor effect, pointing to the importance of innate immune response in the antitumor activity mediated by 1,25(OH)2D3. Moreover, in line with the reduced number of lung metastatic foci, non-adherent cells from tumor-bearing lungs of mice treated with aerosolized 1,25(OH)2D3 showed an increased cytotoxic activity against MC-38 tumor. The fact that we did not observed an increase number of effector cells after 1,25(OH)2D3 exposure, but instead, and upregulation of the CD69 marker, possibly suggests that 1,25(OH)2D3 does not have the ability to increase the recruitment of immune cells to the tumor bed but it can enhance their activation.

An enhanced ability to lyse YAC-1 cells was also observed in vitro after co-culture of 1,25(OH)2D3 pre-treated AM with NK cells.

Although it is well known that, at least in part, NK cell cytotoxic activity is regulated through the interaction with macrophages [30, 43], it is not possible to exclude that 1,25(OH)2D3 may exert a direct influence on these immune cells in vivo. However, 1,25(OH)2D3 biological activity on NK cells is far to be completely elucidated and, based on the available data, it is difficult to draw final conclusions. Indeed, in the literature it is possible to find many examples about how 1,25(OH)2D3 can promote or dampen NK cell cytotoxicity. It is plausible to hypothesize that vitamin D may act tuning the immunomodulatory properties of other factors present in the immune microenvironment in which NK cells are exerting their function and, therefore, its biological effect might be defined as “context-dependent”. For instance, 1,25(OH)2D3 supplementation has been described to increase NK activity in control mice but not in obese mice and in patients on hemodialysis [44, 45]. Accordingly, it has been observed that hypovitaminosis D, quantified measuring serum 25-hydroxyvitamin D3 and D2 levels, negatively impacted on the ability of NK cells to mediate antibody-mediated cellular cytotoxicity against colon carcinoma cells [46]. Moreover, 1,25(OH)2D3 is able to stimulate the expression and the activity of different isoforms of protein Kinase C (PKC) and of N-alpha-benzyl-oxycarbonyl-L-lysine thiobenzyl ester (BLT) esterase, two important proteins positively involved in NK cell function [47]. On the contrary, other reports depicted a completely different scenario, where 1,25-(OH)2D3 treatment was linked to an inhibitory effect on NK cells [48]. For example, in a rat experimental model of 1,25-(OH)2D3 deprivation, it has been reported that NK cell cytotoxic activity was not affected by 1,25-(OH)2D3 deficiency but restoration of 1,25-(OH)2D3 levels by diet supplementation reduced the function of these immune cells [49]. A similar situation was also observed in human setting. Indeed, in vitro experiments suggested that 1,25(OH)2D3 can affect human NK activation in a dose-dependent manner [50]. In addition, it appears that calcitriol impairs the capability of NK cells to secrete chemotactic molecules [51]. More recently, in a study conducted on NK cells obtained from women with recurrent pregnancy loss usually characterized by higher cytotoxic potential compared to normal controls, it was demonstrated that in vitro treatment with 1,25(OH)2D3 was crucial to determine a NK cell function dampening. The Authors found that 1,25(OH)2D3 was able to decrease activation and degranulation markers and cytokine production paralleled by an increase of inhibitory markers [52]. Collectively, these findings make us to understand that the full comprehension of the biological properties of Vitamin D on immune cells, especially NK cells, needs to be extensively explored. As already mentioned, innate immune cells are extremely susceptible to environmental stimuli and, probably, Vitamin D intervenes modulating the signaling pathways triggered by those factors. Therefore, due to the huge heterogeneity of the immumodulatory molecules, it might be difficult or, even, erroneous trying to categorize as immunostimulatory or immunosuppressive this pleiotropic hormone.

As previously mentioned, we showed that 1,25(OH)2D3 can activate AM. However, a randomized trial, revealed that orally supplemented vitamin D3 minimally affects AM gene expression [53], supporting that macrophages within the lung are poorly responsive to circulating 1,25(OH)2D3. This observation could be explained by the fact that the concentration of 1,25(OH)2D3 reaching the lungs is not enough to produce any biological effect on AM, sustaining our idea that local administration of 1,25(OH)2D3 via aerosol might represent an effective strategy to improve AM activity against cancer. The possibility to deliver 1,25(OH)2D3 and 25(OH)D3 by inhalation has been already investigated [29] revealing the efficacy of this route in promoting the maturation of immature lungs in neonatal rats. Moreover, studies by Hansdottir and colleagues reported that 1,25(OH)2D3 or 25(OH)D3 administration is a strategy to decrease harmful inflammatory response by impairing the secretion of inflammatory chemokines in the lung, while maintaining the immunosurveillance against pathogens [54, 55].

Beyond the ability to activate innate immune effectors, it is widely thought that 1,25(OH)2D3 plays an important role in the modulation of the inflammation and mechanistic studies have shown that 1,25(OH)2D3 may inhibit the synthesis of pro-inflammatory mediators un several epithelial cells and also in cancer cells [7, 56–59]. Thus, even if not investigated in this study, a reduction of tumor-driven inflammation in the lung can also concur to the observed 1,25(OH)2D3 impairment of tumor growth.

We focused on the potential anti-metastatic effect of local administration by aerosol of 1,25(OH)2D3, providing data pointing to 1,25(OH)2D3 as therapeutic agent. However, it remains fundamental to exactly define the mechanisms triggered by 1,25(OH)2D3 on cancer and/or immune cells.

Low 25(OH)D3 status is also associated with other lung disease, particularly with increased susceptibility to upper respiratory tract infections [13–18]. Currently, the most relevant of the viral infections that is afflicting the whole world, and for which there are currently no effective therapies, is the COVID-19 pandemic. The role of 1,25(OH)2D3 and 25(OH)D3 status in COVID-19 patients is a matter of debate and several studies have attempting to define whether the relative 1,25(OH)2D3 status of populations can influence outcome of COVID-19. 1,25(OH)2D3 levels are lower in hospitalized COVID-19 patients compared to population-based controls. In a cross-sectional analysis across Europe, COVID-19 mortality was significantly associated with 25(OH)D3 status in different populations, however, this observation has not been confirmed by other studies [60, 61]. One theory is that 1,25(OH)2D3 can contribute in controlling viral replication and reducing the hyper-inflammation. Indeed, immune dysregulation, ultimately leading to hyper-inflammatory cytokine storm, is a key feature observed in patients with severe COVID-19 [62]. Vitamin D2 or Vitamin D3 supplementation can be a relatively cheap and safe strategy to restore immune balance in such risk groups [63, 64].

Several therapeutic agents have been explored for inhalation delivery for the treatment of different tumors, including chemotherapeutic agents, cytokines, antisense oligonucleotides and monoclonal antibodies, demonstrating the feasibility of aerosol delivery. Beyond reaching high local concentration in the airways, the direct local delivery of 1,25(OH)2D3 by aerosolization could avoid toxic effect that, although rare, may manifest as dehydration and result in high calcium uptake. The severe hypercalcemia represents a common side effect related to the systemic route administration that can limit the application in clinics [65, 66]. Accordingly, no effect of 1,25(OH)2D3 or 25(OH)D3 inhalation on either serum calcium levels has been observed [29]. However, is necessary to perform further study to compare the anti-tumor effectiveness of oral vs. aerosol supplementation and to evaluate calcium homeostasis to define whether delivery through aerosolization can overcome such limitations.

Another advantage of local delivery is represented by the possibility to potentially overcome genotype-dependent factors, as variations in the expressions of genes involved in absorption and systemic circulation of free 25(OH)D3 that are reported to affect finally 1,25(OH)2D3 activity [67–69]. Moreover, several lifestyle-related factors (i.e., body mass index, physical activity, smoking, diet, and use of other supplements) have been reported to influence circulating 25(OH)D3 levels [70] and could also impair/reduce the anti-cancer efficacy of systemically-administered 1,25(OH)2D3 or 25(OH)D3.

## Conclusions

Together, our study reveals that aerosolization of 1,25(OH)2D3 can be as a feasible clinical therapeutic procedure to locally activate lung immunity and can be combined with conventional strategies in the treatment of lung cancer and metastases.

## Supporting information

S1 Fig Gating strategies used to analyze BAL suspensions. (DOCX)

## Abbreviations

1,25(OH)2D3: 1,25-dihydroxyvitamin D3
25(OH)D3: 25-hydroxyvitamin D3
AM: alveolar macrophages
BAL: bronchoalveolar lavage
ON: overnight
TME: tumor microenvironment
VDR: vitamin D receptor
