# Supplementary material for: Aerosol 1,25-dihydroxyvitamin D3 supplementation: A strategy to boost anti-tumor innate immune activity
Source: PLoS One. 2021 Mar 29;16(3):e0248789. doi: 10.1371/journal.pone.0248789 (PMC8007042; doi:10.1371/journal.pone.0248789)
Supplement: S1 Fig — (DOCX) [file pone.0248789.s001.docx]

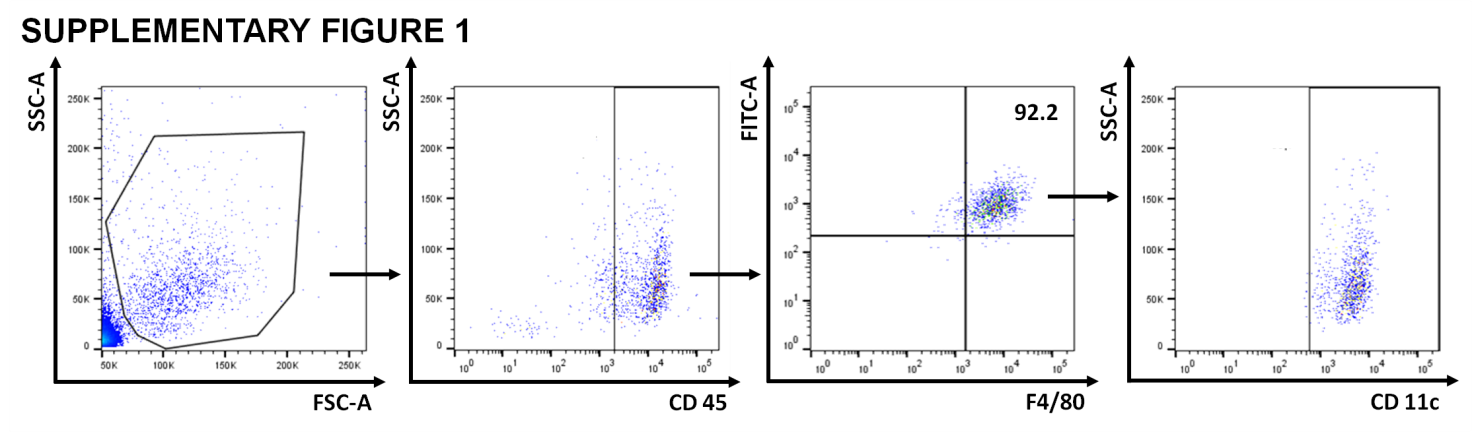


**S1 Fig.** **Gating strategies used to analyze BAL suspensions**. Analysis revealed that alveolar macrophages (AMs) were more than 90% of cells. AMs were identified as F4/80+FL-1+CD11c+ cells among CD45+ cells (FL-1 channel was reserved for the assessment of autofluorescence).
